# Fading authority, rising depression: occupational identity and mental health among China’s retired danwei leaders

**DOI:** 10.3389/fpsyt.2025.1663695

**Published:** 2025-11-24

**Authors:** Xubin Zhang, Yang Yang, Li He, Zihan Wang

**Affiliations:** 1 School of Philosophy, Zhongnan University of Economics and Law, Wuhan, China; 2 School of Politics and International Studies, Central China Normal University, Wuhan, China; 3 School of Country and Region Studies, Beijing International Studies University, Beijing, China

**Keywords:** Danwei leaders, retirement, depression, physical health, social relationships, occupational identity, older adults

## Abstract

Late-life depression is a significant public health concern, yet its relationship with occupational identity in retirement remains underexplored. This study investigates whether retired *Danwei* leaders—individuals who formerly held leadership roles within China’s state-affiliated work units—experience higher levels of depressive symptoms compared to other retirees. Using nationally representative data from the 2016 and 2018 waves of the China Longitudinal Aging Social Survey (CLASS), we apply mixed-effects regression models to examine the association between occupational identity and depression, along with the underlying mechanisms. The results indicate that retired Danwei leaders exhibit significantly higher levels of depression, and this association appears to be linked to declining physical health and diminished interpersonal relationships following retirement. These findings offer new empirical evidence on the mental health implications of high-status occupational identity in post-retirement life and underscore the need for targeted mental health interventions and social support strategies for this vulnerable subgroup of older adults in China.

## 1 Introduction

Depression is a common mental disorder that adversely affects both physical and mental health. According to the World Health Organization (1), Approximately 4% of the global population suffers from depression, with the prevalence rising to 5.9% among adults aged 70 and older. As one of the countries experiencing rapid population aging, China faces a particularly serious challenge, with an estimated depression prevalence of around 20% among its older adult population (2). In China, the prevalence of depression among older adults is particularly high, estimated at around 20% (2). Scholars have attributed this pattern to several social and cultural factors, including the pressures of rapid urbanization (3), changing family structures and declining intergenerational support (4), and the low utilization of mental health services, largely due to limited accessibility and persistent stigma (5). These contextual factors provide important background for understanding the mental health challenges faced by older Chinese adults. While numerous studies have indicated that the psychological distress experienced by older adults often stems from role loss—specifically, the negative psychological impact resulting from the loss of work-related roles during retirement—this effect is not uniform across occupational categories. The psychological consequences of role loss vary considerably depending on the nature of the occupation and its hierarchical status (6).

The Danwei (work unit) system, a defining feature of urban China since the 1950s, has functioned not only as a place of employment but also as a socio-institutional structure that organizes multiple aspects of urban residents’ lives, including housing, health care, pensions, political participation, and social activities (7). Although market reforms have weakened some of its economic functions, the institutional and symbolic influence of the Danwei persists to this day.

Within this system, Danwei leaders held high-ranking positions with substantial administrative authority, welfare entitlements, and symbolic prestige. Upon retirement, however, they face a profound loss of institutional authority and occupational identity, creating a sharp contrast between pre-retirement privilege and post-retirement vulnerability. This discontinuity makes them a highly representative case for examining how occupational identity shifts affect mental health. Comparable occupational groups in other contexts include senior white-collar professionals and top-level managers in large bureaucracies and the public sector (8).

Studying retired Danwei leaders is thus particularly illuminating: their typicality grants the study broader international significance by highlighting general mechanisms—loss of role-based authority, diminished access to institutional resources, and contraction of workplace-centered social networks—that may similarly shape late-life psychological outcomes among retirees from other strongly institutionalized employment systems (e.g., civil service, state-owned enterprises, military/police, or large corporate organizations) worldwide.

Despite the prominence of such roles, there remains a lack of research on the relationship between retirees’ former occupational status and depressive symptoms in later life, and understanding the mental health outcomes of retirees from specific occupational hierarchies is essential for informing targeted intervention strategies. To address this gap, the present study focuses on retired *Danwei* leaders in China, examining the association between their prior occupational status and depression, as well as the mechanisms that mediate this relationship.

### 1.1 Occupational identity of retired *Danwei* leaders and depression levels

The relationship between retirees’ former occupational identity and their levels of depression remains a topic of debate within the academic community. Some scholars argue that there is no significant association between prior occupational identity and post-retirement depression (9). In addition, several studies suggest that retirement may reduce psychological stress, especially for individuals leaving high-demand or high-stress occupations (10, 11), and may lead to greater life satisfaction (12, 13).

Conversely, other studies contend that former occupational identity has a significant negative impact on retirees’ mental health, indicating that psychological well-being in later life is closely tied to the roles individuals held during their careers. This perspective is supported by role theory, which provides a theoretical framework for understanding how individuals’ attachment to occupational roles affects their psychological well-being. Role theory emphasizes the concept of role centrality, which distinguishes between core and peripheral roles and assesses individuals’ degree of involvement in them (14). Roles with high centrality typically receive more personal investment and social recognition. As such, withdrawal from these roles can severely affect self-concept and identity (15). Compared to roles of lower centrality, retirement from high-centrality roles entails not only the loss of core work functions but also a decline in social connections, professional affirmation, and identity reinforcement (16, 17), all of which may contribute to increased role loss and subsequent mental health challenges (18).

In the Chinese context, *Danwei* leaders represent a prototypical high-centrality role. Upon retirement, these individuals not only lose their social networks and occupational prestige but also their influence and decision-making authority, potentially heightening the risk of psychological distress (19). Moreover, the cumulative stress experienced during their professional careers (20, 21) may carry over into retirement, further exacerbating mental health issues. Taken together, these factors suggest that individuals retiring from high-status positions such as *Danwei* leadership may be particularly vulnerable to depression in later life.

Given the high centrality of occupational roles such as *Danwei* leadership and their potential impact on post-retirement mental health, the following hypothesis is proposed: The loss of occupational identity among retired *Danwei* leaders is associated with elevated levels of depression. (Hypothesis 1)

### 1.2 The mediating role of physical health status

Health is not merely the absence of disease or infirmity, but a state of complete physical, mental, and social well-being (22). A growing body of research suggests that physical health plays a protective role against depressive symptoms (23, 24). Accordingly, it is reasonable to consider physical health status as a potential pathway linking the occupational identity of retired *Danwei* leaders to their levels of depression.

Empirical studies have shown that senior managers tend to report better overall health than general workers (26, 27). One possible explanation is that individuals in high-ranking positions, despite experiencing chronic work-related stress and demanding responsibilities, often adopt more proactive health behaviors and structured lifestyles during their careers. These behaviors—including regular schedules and ongoing health monitoring—are associated with improved physical health and a lower risk of depression (25, 28).

However, upon retirement, *Danwei* leaders may find it difficult to maintain the same level of physical activity and health management practices. Compared to other retirees, this disruption may result in fewer health checkups, reduced physical engagement, and a gradual decline in physical condition. A deterioration in physical health can undermine individuals’ sense of self-integrity and self-worth, thereby increasing their vulnerability to negative emotions, particularly depression (29, 30).

Hence, the following hypothesis is proposed: Physical health status mediates the relationship between retired *Danwei* leader status and depression levels. (Hypothesis 2)

### 1.3 The mediating role of interpersonal relationships

The quality and supportiveness of social relationships are widely recognized as key factors in maintaining mental health. Previous research has demonstrated that strong social ties play a protective role in both the prevention and treatment of depression (31, 32). Based on this understanding, interpersonal relationships may serve as a potential mediating pathway in explaining depressive symptoms among retired *Danwei* leaders.

Empirical studies have shown that the size of one’s social network (33) and the level of perceived social support (34) are significant predictors of depression in older adults (35). During the transition from work to retirement, *Danwei* leaders—compared to retirees from other occupational backgrounds—are more likely to experience disruptions in their social relationships and reduced frequency of social interactions. These changes may increase feelings of loneliness (36), thereby raising the risk of depression (37).

Furthermore, Social Identity Theory posits that individuals derive a sense of self from their group memberships, and a loss of social identity may lead to emotional vulnerability. Research suggests that individuals with diminished social identity are more susceptible to depression (38, 39). Retired Danwei leaders, who previously occupied positions of high social recognition and influence, may suffer a sharper loss of social identity after retirement, experiencing a diminished sense of belonging, purpose, and social relevance (31). This loss can lead to lower levels of well-being (40) and weakened social identity (41), thereby increasing the risk of mental health problems such as depression and anxiety (42).

Hence, the following hypothesis is proposed: Changes in social relationships mediate the relationship between retired *Danwei* leader status and depression levels. (Hypothesis 3)

### 1.4 Objectives and analytical strategy

Despite increasing attention to mental health in later life, there remains a lack of research on the mechanisms linking occupational identity in retirement to depression—particularly among individuals from specific occupational hierarchies. This study aims to fill this gap by pursuing the following three objectives:

1. to replicate and extend existing research on the association between occupational identity in retirement and depression, with a specific focus on retired *Danwei* leaders;
2. to examine the parallel mediating roles of physical health and social relationships in explaining this association;
3. to test the moderating effect of willingness to participate in social activities, in order to better understand the conditions under which occupational identity affects depression among retired *Danwei* leaders.

By employing multiple measurement models, this study seeks to provide a more nuanced understanding of the relationship between occupational identity and depression in later life.

## 2 Methods

### 2.1 Data

This study draws on data from the 2016 and 2018 waves of the China Longitudinal Aging Social Survey (CLASS), conducted by the China Survey and Data Center at Renmin University of China. The choice of this time frame was based on two considerations. First, information on the key mediating variables was collected only in these two waves. Second, the survey instruments and sampling design were highly consistent across 2016 and 2018, minimizing potential bias from measurement inconsistencies. The survey employed a stratified, multi-stage probability sampling design, using counties as the primary sampling units and residential/village committees as the secondary sampling units, thereby ensuring broad representativeness within China’s rapidly evolving demographic landscape. Based on the relevance of questionnaire items, this study focuses specifically on retired Danwei leaders. After excluding cases with missing values, refusals, or clearly erroneous responses on key variables, the final analytic sample comprised 3,102 individuals.

### 2.2 Measure

#### 2.2.1 Dependent variable

The dependent variable in this study is the level of depression, measured using items adapted from the Center for Epidemiologic Studies Depression Scale (CES-D), originally developed by Radloff (1977). The depression score was derived from nine questions in the CLASS survey assessing respondents’ mood over the past week, including items such as “Have you felt very sad in the past week?” These questions capture various dimensions of psychological well-being, including negative affect, loneliness, life satisfaction, sleep quality, perceived self-worth, and identity.

Each item was rated on a three-point scale: “never” (1), “sometimes” (2), and “often” (3). The total score ranges from 9 to 27, with higher scores indicating a greater tendency toward depression and poorer mental health.

#### 2.2.2 Independent variable

The independent variable in this study is the status of retired Danwei leaders, as identified through respondents’ answers to the CLASS survey question: “What was your occupation before you stopped working for income?” Individuals who reported their former occupation as “national, enterprise, or institutional leaders” were coded as 1, representing retired Danwei leaders. Those who selected “professional technicians,” “general office staff,” or “general staff in commerce, service, or manufacturing sectors” were coded as 0, indicating retirees who did not hold Danwei leadership positions.

#### 2.2.3 Control variables

In selecting control variables, this study draws on the work of Wang et al. (43) and includes demographic characteristics such as gender, age, ethnicity, marital status, religious beliefs, and whether living with children. It also incorporates health-related variables, including basic functional ability and ability to perform daily activities, as well as social support, which serves as an indicator of social capital.

#### 2.2.4 Instrumental variable

To evaluate the robustness of the association between retired work-unit identity and depression, this study employs “whether the pre-retirement employer belonged to the public sector” as an instrumental variable to address potential endogeneity. This variable satisfies both the relevance and exogeneity conditions.

In terms of relevance, leadership positions in China’s public sector organizations (party and government agencies, public institutions, and state-owned enterprises) are standardized and associated with explicit administrative ranks, whereas management positions in the non-public sector are more flexibly defined (44). Thus, pre-retirement sector type is a strong predictor of whether an individual held a leadership position.

In terms of exogeneity, any direct effect of sector type on depression would primarily operate through cumulative work environment effects, social support structures, and family patterns. However, these influences are mitigated by controlling for health, social capital, and demographic covariates. Institutionally, leaders in both public and non-public sectors enjoyed similar high status and resource advantages prior to retirement (45), and the core challenge they faced was identity transformation and status decline after retirement. Sector type therefore serves mainly as an identifier of “work-unit leadership” and does not directly affect depression.

The instrumental variable was constructed from respondents’ answers to the survey item on “type of pre-retirement work unit.” Those who reported “government departments/party and government agencies/people’s organizations,” “public institutions,” “state-owned or state-controlled enterprises,” “collective enterprises,” or “military” were coded as 1 (public sector before retirement). All other responses were coded as 0 (non-public sector before retirement).

#### 2.2.5 Mediating variables

Building upon the preceding discussion and drawing on previous studies (31, 32), this study conceptualizes the impact of retired Danwei leaders’ occupational identity on depression through two primary pathways: changes in physical health and changes in social relationships.

The extent of change in physical health is measured using a single-item scale, with response options ranging from “basically no change,” “very little change,” and “moderate change,” to “significant change,” coded as 1, 2, 3, and 4, respectively. Higher scores reflect greater deterioration in physical health after retirement compared to pre-retirement.

Changes in social relationships are assessed using three items that evaluate perceived changes in relationships with family, former colleagues, and friends or neighbors. Each item follows the same four-point response scale used for physical health. The total score is calculated by summing the three items, with higher scores indicating greater perceived disruption in social relationships after retirement.

#### 2.2.6 Moderating variable

Given that retired *Danwei* leaders’ subjective attitudes toward society may influence their mental well-being, this study incorporates “willingness to participate in social activities” as a moderating variable.

This variable is derived from two items in the CLASS survey: “If given the opportunity, I am willing to participate in some work of the village/residential committee” and “I often think about doing more for society.” Responses to both items are rated on a five-point Likert scale: “completely disagree,” “somewhat disagree,” “neutral,” “somewhat agree,” and “completely agree,” coded from 1 to 5, respectively. Higher scores reflect a stronger willingness to engage in social activities.

## 3 Results

### 3.1 Descriptive statistics

**Table 1** presents the descriptive statistics of the sample. The overall level of depression in the sample was relatively high, with a mean score of 14.76. Among all participants, retired Danwei leaders accounted for 19.21%. Regarding other characteristics, the average age of the sample was 69.94 years, and 54.4% were male, indicating a reasonable and balanced gender distribution. Within this retired population, the vast majority were of Han ethnicity and reported no religious affiliation. Approximately 82.4% were married, and 38.23% lived with their children. On the whole, the sample exhibited relatively poor basic and instrumental activities of daily living, while the level of social support was below the average range.

**Table 1:** Variable definitions and descriptive statistics.

Variable	Variables defination	Obs	Mean	Std. Dev.	Min	Max
depression	Each question is graded on a scale of 1 to 3 (1= never; 2= sometimes, 3= often).	3102	14.76	3.081	9	26
work	yes=1, no=0	3102	.192	.394	0	1
sex	male=1, female=0	3102	.544	.498	0	1
age	age of sample	3102	69.974	7.412	60	100
nation	han ethnicity=1, non-han ethnicity=0	3102	.974	.16	0	1
marriage	marriage=1, unmarried/divorced/widowed/cohabitation =0	3102	.834	.372	0	1
religion	yes=1, no=0	3102	.071	.257	0	1
cohabitation	yes=1, no=0	3102	.382	.486	0	1
adl	yes=1, no=0	3102	.134	.341	0	1
iadl	yes=1, no=0	3102	.252	.434	0	1
social support	The value is proportional to the degree of social support, assigned from 1 to 10	3102	4.662	1.868	0	10

### 3.2 Baseline regression

**Table 2** presents the results of the baseline regression models. Model 1 reports the regression results of the key explanatory variable—retired Danwei leader status—on depression levels. Model 2 adds controls for demographic characteristics, while Model 3 further includes variables related to health awareness and social capital. The interpretation of results is based primarily on Model 3.

**Table 2:** Baseline regression results.

	(1)	(2)	(3)
	Depression	Depression	Depression
work	0.510***	0.604***	0.609***
	(0.151)	(0.217)	(0.219)
sex		0.185	0.165
		(0.174)	(0.173)
age		0.001	0.001
		(0.001)	(0.002)
nation		1.890***	1.796***
		(0.449)	(0.436)
marriage		-4.312***	-4.060***
		(0.282)	(0.284)
religion		-0.035	-0.138
		(0.331)	(0.319)
cohabitation		-6.655***	-6.700***
		(0.325)	(0.327)
adl			1.444***
			(0.280)
iadl			0.298
			(0.209)
socialsupport			0.017
			(0.052)
_cons	14.624***	18.757***	18.322***
	(0.070)	(0.549)	(0.601)
lns1_1_1:			
_cons	0.533***	1.031***	-0.958
	(0.154)	(0.037)	(3.552)
lns1_1_2:			
_cons	1.161***	1.442***	1.434***
	(0.013)	(0.031)	(0.038)
atr1_1_1_2:			
_cons	-0.516***	-0.395***	-0.177*
	(0.071)	(0.045)	(0.092)
lnsig_e:			
_cons	-1.580***	-3.482***	-3.499***
	(0.490)	(0.567)	(0.598)
N	3102	3102	3102

Robust standard errors are reported in parentheses; *, **, *** denote significance at the 10%, 5%, 1% levels, respectively.

The findings indicate that, after controlling for a range of covariates, retired Danwei leaders exhibit significantly higher levels of depression compared to retirees from other occupational backgrounds. Specifically, their probability of depression is 41% higher, and this result is statistically significant at the 1% level. These results suggest that holding a Danwei leadership identity in retirement is significantly associated with increased depressive symptoms.

The control variables also yield informative insights. At the demographic level, gender, religious belief, and number of children are not significantly associated with depression levels. However, older individuals tend to report higher levels of depression. Members of the Han ethnic majority exhibit significantly higher depression levels than ethnic minorities. Being married and having fewer children are both associated with significantly lower depression levels.

Regarding health status, individuals without disabilities in basic and daily functional activities show significantly lower levels of depression. In terms of social capital, retirees who report greater social support and maintain broader social networks—such as frequent contact with family, relatives, and friends, and receiving help from others—tend to experience lower levels of depression.

### 3.3 Robustness checks

#### 3.3.1 PSM Test

The results above suggest that holding the identity of a retired Danwei leader is significantly associated with elevated levels of depression. To enhance the scientific validity and credibility of these regression findings, this study conducted robustness checks using nearest neighbor matching, kernel matching, and radius matching methods.

**Table 3** presents the results of the covariate balance test. After matching, the standardized differences of most covariates fall below 10%, and the differences between the treatment and control groups are statistically insignificant. This indicates that the propensity score matching (PSM) procedure has effectively reduced selection bias in the sample.

**Table 3:** Balance test.

Variable	Unmatched/Matched	Treated	Control	Bias%	Reduct bias%	T-value	P-value
sex	U	0.639	0.521	24.100		5.220	0.000
	M	0.639	0.639	0	100.000	0.000	1.000
age	U	71.227	69.676	20.800		4.610	0.000
	M	71.140	71.034	1.4	93.100	0.240	0.087
nation	U	0.980	0.972	5.1		1.070	0.286
	M	0.980	0.987	-4.400	13.500	-0.900	0.367
marriage	U	0.861	0.828	9.1		1.950	0.051
	M	0.862	0.874	-3.300	64.400	-0.600	0.549
religion	U	0.037	0.079	-18.100		-3.600	0.000
	M	0.035	0.037	-0.700	96.000	-0.160	0.877
cohabitation	U	0.378	0.383	-1.200		-0.270	0.788
	M	0.379	0.361	3.8	-211.100	0.660	0.509
adl	U	0.119	0.138	-5.500		-1.190	0.233
	M	0.118	0.123	-1.500	72.700	-0.270	0.789
iadl	U	0.193	0.266	-17.400		-3.690	0.000
	M	0.192	0.185	1.6	90.700	0.300	0.767
social support	U	4.765	4.638	7		1.500	0.134
	M	4.774	4.784	-0.600	92.100	-0.100	0.919

**Table 4** reports the average treatment effect on depression levels among retired Danwei leaders. The nearest neighbor matching results show that, compared to retirees without Danwei leadership experience, retired Danwei leaders exhibit depression scores that are 0.404 points higher, significant at the 5% level—consistent with the baseline regression results. Similar findings are obtained using kernel matching and radius matching, further confirming the robustness of the main conclusions.

**Table 4:** Average treatment effect for the treatment group.

Matching method	Treatment group	Control group	ATT	Bootstrap standard error	T-value
Nearest Neighbor Matching	15.108	14.704	0.404**	0.196	2.31
Kernel Matching	15.098	14.680	0.400***	0.138	3.03
Radius Matching	15.108	14.704	0.404**	0.196	2.31

*, **, *** represent significance at the 10%, 5%, and 1% levels, respectively; the standard errors after matching in the fifth row are obtained from 500 bootstrap calculations.

#### 3.3.2 Entropy balancing test

To verify the robustness of the baseline regression, this study further employed the entropy balancing method (Entropy Balancing), which is considered more accurate for handling high-dimensional data. This method, first proposed and applied by Hainmueller (46), assigns optimal weights to covariates by constraining the first, second, and third moments of the covariates in the treatment and control groups to achieve balance. In doing so, the weighted distributions of the treatment and control groups are made as similar as possible, thereby attaining exact matching. A simple regression is then conducted on the matched sample, and the coefficient of the explanatory variable represents the average treatment effect.

In this study, the treatment group comprised retired Danwei leaders, while the control group consisted of non-Danwei retirees. **Tables 5**, **6** report the means, variances, skewness, and standardized differences of covariates between the treatment and control groups before and after entropy balancing. The results show that covariate differences existed between the two groups prior to entropy balancing; however, after assigning the optimal weights, the covariates were largely balanced across groups. **Table 7** presents the regression results based on entropy balancing. It can be observed that, after entropy balancing, compared with retirees from other occupational categories, retired Danwei leaders had a 38.7% higher likelihood of depression, which was statistically significant at the 5% level. This conclusion is consistent with the previous PSM results and provides further support for Hypothesis 1.

**Table 5:** Means, variances, skewness, and standardized differences between the treatment and control groups before entropy balancing.

Variable	Treatment group			Control group			Standardized difference
	Mean	Variance	Skewness	Mean	Variance	Skewness	
sex	0.639	0.231	-0.58	0.521	0.25	-0.085	0.246
age	71.227	57.201	0.705	69.676	53.961	0.757	0.205
nation	0.98	0.02	-6.833	0.972	0.027	-5.73	0.055
marriage	0.861	0.12	-2.084	0.828	0.143	-1.735	0.096
religion	0.037	0.036	4.912	0.079	0.073	3.121	-0.223
cohabitation	0.378	0.235	0.505	0.383	0.237	0.479	-0.012
adl	0.119	0.105	2.352	0.138	0.119	2.103	-0.057
iadl	0.193	0.156	1.556	0.266	0.195	1.061	-0.184
social support	4.765	3.121	-0.101	4.638	3.574	0.197	0.072

**Table 6:** Means, variances, skewness, and standardized differences between the treatment and control groups after entropy balancing.

Variable	Treatment group			Control group			Standardized difference
	Mean	Variance	Skewness	Mean	Variance	Skewness	
sex	0.639	0.231	-0.58	0.639	0.231	-0.58	0
age	71.227	57.201	0.705	71.226	60.366	0.561	0
nation	0.98	0.02	-6.833	0.98	0.02	-6.832	0
marriage	0.861	0.12	-2.084	0.861	0.12	-2.084	0
religion	0.037	0.036	4.912	0.037	0.036	4.909	0
cohabitation	0.378	0.235	0.505	0.378	0.235	0.505	0
adl	0.119	0.105	2.352	0.119	0.105	2.351	0
iadl	0.193	0.156	1.556	0.193	0.156	1.556	0
social support	4.765	3.121	-0.101	4.765	3.579	0.236	0

**Table 7:** Mixed-effects regression analysis based on entropy balancing.

	Depression
work	0.387**
	(0.151)
sex	0.119
	(0.147)
age	0.022**
	(0.010)
nation	1.385***
	(0.464)
marriage	-0.619***
	(0.233)
religion	-0.379
	(0.300)
cohabitation	-0.142
	(0.155)
adl	0.467*
	(0.251)
iadl	0.136
	(0.177)
social support	-0.100**
	(0.042)
_cons	12.723***
	(0.917)
lns1_1_1:	
_cons	0.314
	(0.000)
lns1_1_2:	
_cons	-11.918
	(0.000)
atr1_1_1_2:	
_cons	-0.903
	(0.000)
lnsig_e:	
_cons	1.000
	(0.000)
N	3102

*, **, *** indicate significance at the 10%, 5%, and 1% levels, respectively; the standard errors in column 5 after matching were obtained through 500 bootstrap replications.

#### 3.3.3 Alternative models

To further assess the robustness of the regression results, this study employs an ordinary least squares (OLS) regression model as a substitute for the mixed-effects model. The results are presented in **Table 8**. Column (1) shows that, after controlling for demographic characteristics, health status, and social capital variables, the depression level of retired Danwei leaders is 0.387 points higher than that of non-Danwei leaders, and this effect is statistically significant at the 1% level. This finding is consistent with the results from the mixed-effects model, thereby confirming the robustness of the primary conclusion.

**Table 8:** Alternative models.

	(1)	(2)	(3)
	Depression	Depression	Depression
work	0.387***	0.026***	0.026***
	(0.129)	(0.009)	(0.009)
sex	0.101	0.007	0.007
	(0.113)	(0.008)	(0.008)
age	0.032***	0.002***	0.002***
	(0.008)	(0.001)	(0.001)
nation	1.094***	0.077***	0.078***
	(0.304)	(0.022)	(0.022)
marriage	-0.610***	-0.041***	-0.041***
	(0.175)	(0.012)	(0.012)
religion	-0.446**	-0.031**	-0.031**
	(0.221)	(0.015)	(0.015)
cohabitation	-0.280**	-0.019**	-0.019**
	(0.123)	(0.008)	(0.008)
adl	0.783***	0.051***	0.051***
	(0.175)	(0.011)	(0.011)
iadl	0.149	0.009	0.010
	(0.131)	(0.009)	(0.009)
social support	-0.112***	-0.008***	-0.008***
	(0.030)	(0.002)	(0.002)
_cons	12.382***	2.522***	2.527***
	(0.662)	(0.045)	(0.045)
			
lnalpha			-22.030
			(0.000)
N	3102	3102	3102
R2	0.042		

Robust standard errors are reported in parentheses; *, **, *** indicate significance at the 10%, 5%, and 1% levels, respectively.

In addition, this study applies Pseudo-Poisson Maximum Likelihood (PPML) regression and Negative Binomial regression as further robustness checks. The results in Column (2) using the PPML model and in Column (3) using the Negative Binomial model are both significantly positive at the 1% level, aligning with the baseline regression results and further reinforcing the reliability of the findings.

#### 3.3.4 Instrumental variable analysis

To address potential reverse causality in the analysis, this study employs an instrumental variable (IV) approach. **Table 9** presents the estimated association between being a retired Danwei leader and depression levels. The first-stage regression results in Column (1) show a significant positive association between having worked in the public sector prior to retirement and the likelihood of being a *Danwei* leader. The F-statistic exceeds the conventional threshold of 10, indicating that the selected instrumental variable is sufficiently strong and not weakly correlated with the endogenous variable.

**Table 9:** Instrumental variable estimation results.

	(1)	(2)
	First-stage regression	Second-stage regression
	work	Depression
IVTYPE	0.152*** (0.014)	
work_hat		0.031* (0.018)
Control	Yes	Yes
_cons	-0.264*** (0.086)	14.655*** (0.640)
lns1_1_1: _cons		-1.079 (1.165)
lns1_1_2: _cons		1.084*** (0.106)
atr1_1_1_2:_ cons		-0.150 (0.000)
lnsig_e: _cons		-20.791*** (0.076)
N	3102	3102
R2	0.063	
	F(1, 3091)=119.10	

Robust standard errors are reported in parentheses; *, **, *** denote significance at the 10%, 5%, 1% levels, respectively.

The second-stage regression results in Column (2) demonstrate that the depression level of retired *Danwei* leaders is 0.031 points higher than that of non-*Danwei* leaders, and this effect is statistically significant at the 1% level. This result aligns with the baseline regression findings and supports the conclusion that *Danwei* leadership identity in retirement is associated with elevated levels of depression.

### 3.4 Mechanism analysis

Building on the above findings, changes in physical health and social relationships before and after retirement may partially explain the elevated depression levels observed among retired *Danwei* leaders. To examine these potential mechanisms, this study adopts the stepwise regression method proposed by Wen et al. (47). The detailed regression results are presented in **Table 10**.

**Table 10:** Mediating effect estimation results.

	(1)	(2)	(3)	(4)
	Body change	Depression	Relationship change	Depression
work	0.315***	0.235	0.811***	0.106
	(0.046)	(0.177)	(0.119)	(0.181)
body change		1.043***		
		(0.053)		
relationship change				0.579***
				(0.022)
sex	0.052	0.127	-0.031	0.196
	(0.036)	(0.138)	(0.093)	(0.141)
age	-0.001	0.004*	-0.002	0.003*
	(0.001)	(0.002)	(0.003)	(0.001)
nation	0.254**	1.298***	0.391	1.390***
	(0.106)	(0.407)	(0.275)	(0.417)
marriage	-0.206***	-2.328***	-0.818***	-2.422***
	(0.053)	(0.197)	(0.134)	(0.201)
religion	0.179***	-0.585**	0.589***	-0.679**
	(0.069)	(0.264)	(0.178)	(0.271)
cohabitation	-0.364***	-3.359***	-1.614***	-3.489***
	(0.035)	(0.110)	(0.082)	(0.111)
adl	0.366***	0.846***	0.637***	0.912***
	(0.056)	(0.216)	(0.145)	(0.221)
iadl	0.222***	0.012	0.060	0.224
	(0.044)	(0.169)	(0.114)	(0.172)
social support	-0.087***	-0.037*	-0.184***	0.012
	(0.008)	(0.020)	(0.019)	(0.015)
_cons		14.527***	6.575***	13.390***
		(0.498)	(0.360)	(0.499)
lns1_1_1:	2.179***	1.208***	0.814***	1.232***
_cons	(0.152)	(0.017)	(0.019)	(0.015)
lnsig_e:	-0.137***	-2.598***	-2.366***	-3.011***
_cons	(0.015)	(0.048)	(0.060)	(0.036)
N	3102	3102	3102	3102

Robust standard errors are reported in parentheses; *, **, *** denote significance at the 10%, 5%, 1% levels, respectively.

Columns (1) and (3) report the effects of *Danwei* leadership status on the two mediating variables—physical health changes and changes in social relationships. Columns (2) and (4) show the results of the baseline regression models after including each mediating variable. The results suggest that the identity of retired *Danwei* leaders influences depression levels through both pathways: deteriorating physical health and weakened social relationships after retirement.

### 3.5 Moderating effect

To verify whether “willingness to participate in social activities” and depression represent distinct constructs, we followed the approach of Obilor and Amadi (48) and conducted correlation tests among all variables using the Pearson correlation coefficient (r). **Table 11** presents the full correlation matrix. The results show that the correlation coefficient between “willingness to participate in social activities” and depression was only 0.032 (although statistically significant, the effect size was extremely small), indicating that there is virtually no substantive linear correlation between the two variables (49).

**Table 11:** Correlation matrix of all variables.

	Depression	Work	Sex	Age	Nation	Marriage	Religion
depression	1						
work	0.053***	1					
sex	0.0130	0.093***	1				
age	0.129***	0.082***	0.065***	1			
nation	0.067***	0.0190	0.0190	0.062***	1		
marriage	-0.094***	0.035*	0.216***	-0.268***	-0.00900	1	
religion	-0.041**	-0.065***	-0.0190	0.00500	-0.127***	0.00900	1
cohabitation	0.00100	-0.00500	-0.092***	0.082***	0.030*	-0.391***	0.031*
adl	0.119***	-0.0210	-0.0230	0.261***	0.0180	-0.170***	0.046**
iadl	0.078***	-0.066***	-0.058***	0.270***	-0.00200	-0.158***	0.074***
social support	-0.070***	0.0270	-0.00300	0.00500	0.0150	0.054***	-0.040**
body change	0.167***	0.121***	0.0260	0.083***	0.035*	-0.053***	0.044**
relationship change	0.151***	0.135***	0	0.043**	0.0180	-0.031*	0.042**
will	0.032*	0.043**	0.0180	0.00900	0.00900	0.0140	-0.00600
IVTYPE	0.0150	0.206***	0.038**	0.103***	0.038**	-0.0130	-0.0280
urbanization level	-0.130***	-0.064***	-0.102***	0.00200	0.0130	-0.061***	0.00800
	cohabitation	adl	iadl	social support	body change	relationship chnage	will
cohabitation	1						
adl	0.128***	1					
iadl	0.080***	0.362***	1				
social support	0.0280	-0.00500	-0.056***	1			
body change	0.044**	0.171***	0.144***	-0.116***	1		
relationship change	0.0290	0.092***	0.037**	-0.067***	0.588***	1	
will	0.0110	0.0100	-0.0230	0.00200	0.065***	0.101***	1
IVTYPE	0.0270	0.0160	-0.0260	0.00200	-0.0180	0.00200	0.0150
urbanization level	0.066***	0.040**	0.043**	-0.057***	0.084***	0.089***	0.0150
	IVTYPE	urbanization level					
IVTYPE	1						
urbanization level	-0.133***	1					

*, **, *** indicate significance at the 10%, 5%, 1% levels, respectively.

The regression results of the moderating effect model are reported in **Table 12**. Column (1) indicates that willingness to participate in social activities, as a moderating variable, exerted a suppressing effect on depression levels. In Column (2), the coefficient of the independent variable was significantly negative at the 1% level, while the interaction term was significantly positive at the 1% level, suggesting that “willingness to participate in social activities” played a significant suppressing role in the relationship between retired Danwei leaders and depression levels.

**Table 12:** Estimation results of moderating effects.

	(1)	(2)
	Depression	Depression
work	0.662***	-0.608***
	(0.209)	(0.206)
will	-1.000***	-0.626***
	(0.000)	(0.042)
work *will		1.944***
		(0.219)
sex	0.165	0.198
	(0.163)	(0.161)
age	0.000	0.000
	(0.000)	(0.000)
nation	1.872***	1.830***
	(0.482)	(0.476)
marriage	-4.199***	-4.214***
	(0.223)	(0.220)
religion	-0.144	-0.133
	(0.313)	(0.309)
cohabitation	-7.000***	-7.000***
	(0.000)	(0.000)
adl	1.423***	1.490***
	(0.255)	(0.252)
iadl	0.352*	0.366*
	(0.199)	(0.197)
social support	0.000	0.000
	(0.000)	(0.000)
_cons	18.764***	18.659***
	(0.518)	(0.513)
lns1_1_1:		
_cons	1.379***	1.366***
	(0.000)	(0.001)
lnsig_e:		
_cons	-20.496***	-20.508***
	(0.000)	(0.001)
N	3102	3102

Robust standard errors are reported in parentheses; *, **, *** indicate significance at the 10%, 5%,1% levels, respectively.

### 3.6 Heterogeneity analysis

Regional disparities largely shape older adults’ health status and access to social resources (50). Accordingly, this study conducted a heterogeneity analysis based on the level of urbanization at the prefecture-city level to examine how regional development influences the depressive symptoms of retired Danwei leaders. Drawing on Northam’s (1979) “S-curve” framework of urbanization, the sample was divided into the acceleration stage (30% ≤ urbanization rate < 70%) and the mature stage (urbanization rate ≥ 70%) for separate estimations. The results reported in **Table 13** show that the negative effect of retirement on depression among Danwei leaders was significant only in the mature stage of urbanization. This indicates that retired Danwei leaders in highly urbanized areas exhibit significantly higher levels of depression.

**Table 13:** Results of heterogeneity analysis.

	Acceleration stage	Mature stage
	Depression	Depression
work	0.006	0.670**
	(0.222)	(0.299)
sex	-0.168	0.318
	(0.186)	(0.221)
age	0.003*	0.000
	(0.001)	(0.000)
nation	0.981*	2.104***
	(0.545)	(0.650)
marriage	-0.777***	-4.125***
	(0.289)	(0.289)
religion	-0.473	0.052
	(0.364)	(0.413)
cohabitation	-0.317	-7.000***
	(0.203)	(0.000)
adl	0.925***	1.439***
	(0.294)	(0.338)
iadl	0.399*	0.144
	(0.225)	(0.268)
social support	0.057***	0.000
	(0.014)	(0.000)
_cons	14.443***	18.036***
	(0.628)	(0.692)
lns1_1_1:		
_cons	1.094***	1.378
	(0.021)	(0.000)
lnsig_e:		
_cons	-3.277***	-20.496
	(0.039)	(0.000)
N	1467	1635

Robust standard errors are reported in parentheses; *, **, *** indicate significance at the 10%, 5%, 1% levels, respectively.

## 4 Discussions

This study examined the link between occupational identity and depression in retirement, with a specific focus on retired Danwei leaders. The findings indicate that this group is more likely to experience higher levels of depression, and that this association is partly mediated by declines in physical health and changes in social relationships. In addition, willingness to participate in social activities was found to moderate this relationship, reducing the negative impact of occupational identity on mental health.

In the following sections, we first assess the validity of these results, then discuss possible explanations through health and social pathways, compare our findings with previous research, and highlight the moderating role of social engagement (Section 4.1). We then turn to the broader theoretical and practical implications (Section 4.2), before concluding with limitations and directions for future research (Section 4.3).

### 4.1 Occupational identity and depression

This study finds that the occupational identity of retired Danwei leaders is associated with higher levels of depression compared with other retirees. To further examine the robustness and credibility of this finding, we evaluate the validity of the results from multiple perspectives, including internal validity, external validity, construct validity, and robustness checks using matching and reweighting approaches.

#### 4.1.1 Validity assessment

To ensure the validity and robustness of the findings, this study adopted several methodological strategies.

*Internal validity*. This study adopted multiple strategies in research design and methodological application to ensure the validity and robustness of the findings. First, with respect to internal validity, demographic characteristics, health awareness, and social capital variables were included as controls to systematically reduce potential confounding effects on depression levels. In addition, to address possible endogeneity arising from reverse causality, an instrumental variable approach was employed to strengthen the robustness of the findings and mitigate concerns about endogeneity. Nevertheless, unobserved confounders remain a potential threat. For instance, individual personality traits such as neuroticism may simultaneously influence both retirement decisions and vulnerability to depression, leading to omitted variable bias. Therefore, the internal validity of this study should still be interpreted with caution.

*External validity*. With respect to external validity, the study focused on retired Danwei leaders, a group with typically strong occupational identity and higher social status. The role transition and loss of social identity after retirement may exert particularly pronounced effects on their mental health. As such, the findings can be reasonably generalized to senior managers or professional and technical personnel with similar occupational characteristics, but caution is warranted when extending them to ordinary employees or other occupational groups.

*Construct validity*. In terms of construct validity, depression was measured using the well-established short form of the Center for Epidemiologic Studies Depression Scale (CES-D), ensuring adequate psychometric properties of the measurement instrument. To address potential conceptual overlap between “willingness to participate in social activities” and depression, a correlation test was conducted. The results revealed an extremely low correlation coefficient, indicating no substantive construct confusion and confirming discriminant validity between the two variables.

*Robustness checks.* Finally, multiple robustness checks further reinforced the credibility of the findings. In addition to propensity score matching (PSM) and entropy balancing methods that address sample selection bias, an instrumental variable (IV) approach was employed to mitigate potential reverse causality. The first-stage results confirmed a strong association between prior employment in the public sector and the likelihood of becoming a Danwei leader, with the F-statistic well above the conventional threshold of 10. The second-stage regression showed that retired Danwei leaders reported significantly higher depression levels compared to their non-Danwei counterparts, consistent with the baseline estimates. Furthermore, alternative estimation models—including ordinary least squares, Poisson regression, and negative binomial regression—were applied, and the results remained robust across specifications. Together, these robustness checks confirm that Danwei leadership identity in retirement is significantly associated with elevated depression levels.

Taken together, these strategies substantially bolster the validity and robustness of the findings, increasing confidence in the conclusion that occupational identity plays an important role in shaping the mental health of retired Danwei leaders.

#### 4.1.2 Possible explanations

Several mechanisms may help explain why retired Danwei leaders are more vulnerable to depression.

*Health pathway.* Better physical health is generally associated with lower levels of depression (24). During their careers, Danwei leaders often maintained regular routines and relatively active lifestyles, which benefited their health (28). However, once retired, these health-promoting habits may be disrupted, leading to deterioration in physical health and subsequently increasing depressive symptoms (29).

*Social relationship pathway.* Changes in social networks also play an important role (31). Compared with other workers, Danwei leaders were more likely to build broad social circles and enjoy higher social status during their tenure. Retirement, however, often brings a sharp contraction of networks and a rapid decline in status, which may result in psychological maladjustment, a sense of isolation, and insufficient social support (36, 37). While these losses increase vulnerability to depression, some retirees may attempt to offset them through alternative forms of social engagement—a compensatory mechanism that will be discussed in the next subsection.

*Contextual differences.* Beyond these mechanisms, heterogeneity analysis revealed that retired Danwei leaders in highly urbanized areas displayed significantly higher levels of depression. This may be associated with higher living costs, greater daily stress, and weaker neighborhood support in urban environments (51–53).

Taken together, these mechanisms highlight the vulnerability of retired Danwei leaders to depression. At the same time, they point to the potential importance of protective factors—most notably social activity participation—which will be elaborated in the next subsection.

#### 4.1.3 Moderating effect: social activity participation

As noted above, retirement often leads to a contraction of social networks and diminished social support, increasing the risk of depression among retired Danwei leaders. However, our findings reveal that active participation in social activities can buffer these negative effects. Specifically, willingness to engage in community or volunteer work significantly weakens the adverse association between retired Danwei leadership identity and depression. Retirees who remain socially active report lower levels of depressive symptoms, suggesting that social participation functions as a protective factor for psychological well-being.

This moderating role can be understood through its capacity to sustain a sense of purpose, maintain interpersonal networks, and reconstruct social identities that may otherwise erode after retirement. In this sense, social participation alleviates loneliness and mitigates the identity discontinuity caused by the abrupt withdrawal from institutional authority and prestige.

More broadly, the finding underscores the interplay between structural constraints and individsual agency in shaping late-life mental health. While the occupational identity of Danwei leaders reflects structural dependence on political and organizational authority, their willingness to remain socially active demonstrates agency in adapting to post-retirement life. This interaction indicates that even groups vulnerable to sharp role loss can effectively reduce psychological risks by engaging in meaningful social activities.

Taken together, the moderating effect highlights the importance of fostering social participation opportunities for retired leaders, aligning with broader evidence on active aging and suggesting a promising avenue for intervention in community-based mental health strategies.

#### 4.1.4 Comparison with previous research

This study adds to the long-standing debate on the mental health consequences of retirement. Some prior research has argued that retirement can relieve stress and improve well-being, particularly among those leaving highly demanding or stressful jobs (9, 12, 54, 55). In contrast, other studies emphasize the strong connection between occupational identity and psychological well-being in later life, showing that the loss of high-centrality roles increases vulnerability to depression (16, 18, 56). Our findings align more closely with the latter perspective, demonstrating that retired Danwei leaders—a group with highly central roles—are particularly prone to depression due to the abrupt loss of authority, prestige, and institutional support.

In addition, our results highlight the moderating role of social activity participation, which resonates with international evidence stressing the protective effects of productive and volunteer roles for mental health in later life. Previous studies have shown that maintaining active social engagement helps older adults sustain social integration and a sense of purpose, thereby reducing depressive symptoms (57–59). Consistent with this, we find that willingness to participate in social activities significantly weakens the adverse association between retired Danwei leadership identity and depression. This suggests that even for subgroups facing heightened risks in retirement, active social participation can provide an important pathway to resilience.

In summary, the evidence reveals a potential general pattern: the greater the power, autonomy, and symbolic recognition embedded in one’s occupational role prior to retirement, the greater the psychological costs of losing that role may be afterward. At the same time, our findings indicate that these negative effects are not irreversible; active social participation can partly buffer the psychological risks associated with role loss. In other words, post-retirement mental health outcomes depend not only on the extent of authority and status held before retirement but also on the ability to reconstruct purpose and social connections through engagement afterward. While similar tendencies have been observed in some Western managerial and professional groups, the case of Chinese Danwei leaders underscores the importance of institutional context, showing that post-retirement depression is shaped both by the scope and constraints of pre-retirement authority and by the capacity to maintain social participation afterward.

### 4.2 Implications

This study holds both theoretical and practical significance in understanding how the occupational identity of retired Danwei leaders influences post-retirement depression.

Theoretically, this study contributes to the ongoing debate on the mental health effects of retirement by focusing on an overlooked group—retired Danwei leaders. Chinese retired leaders are particularly typical cases because their pre-retirement roles combined both political authority and socioeconomic privilege. Observing this group offers a clearer lens through which to examine how individuals in positions of high authority experience depression and well-being after retirement. Moreover, because they once occupied positions of hierarchical superiority, these leaders often struggle to adapt to new, more egalitarian social relationships in retirement—precisely the kinds of relationships that are most effective in alleviating psychological distress.

Practically, the findings highlight the need for targeted interventions to mitigate the psychological vulnerabilities of retired leaders. Promoting good health and sustaining interpersonal relationships can be achieved through structured health promotion programs, peer-support networks, and opportunities for continued social engagement. In China, such efforts can be integrated with existing initiatives. For instance, the nationwide *Silver-Age Action* mobilizes retired professionals to contribute to education and community development, while local *Cadre Activity Centers* and *Cadre Universities* offer institutionalized venues for cultural, educational, and recreational engagement. These platforms help maintain social roles, reduce isolation, and alleviate identity loss. Moreover, they are consistent with the *Healthy China 2030* agenda, which emphasizes preventive health care and active aging. Together, these measures illustrate concrete pathways for China and offer valuable insights for global debates on active aging.

### 4.3 Limitations and future directions

Although this study reveals important associations between retirees’ occupational identity and depression, several limitations should be acknowledged.

First, the analysis relies on only two survey waves (2016 and 2018), which constrains the ability to capture long-term dynamics. Mediating variables—physical health and interpersonal relationships—were measured through subjective self-reports at the same time as depression outcomes, making it difficult to establish temporal ordering. As a result, the findings should be interpreted as associations rather than causal effects, and potential reverse causality or unobserved confounding cannot be fully ruled out.

Second, while this study considered both mediating and moderating mechanisms, the scope of variables remains limited. Factors such as economic status, quality of life, or broader community engagement were not included, and these may further shape the relationship between occupational identity and post-retirement depression.

Third, the sample focuses on retired Danwei leaders, whose distinctive pre-retirement authority and social status make them a valuable case for analysis but may limit generalizability to other retiree groups.

Future research could address these limitations by incorporating longer-term longitudinal data, adopting more objective measures of health and social dynamics, and broadening the range of explanatory factors. Comparative studies across occupational hierarchies or institutional contexts would also be valuable in assessing whether the observed patterns are unique to Chinese Danwei leaders or reflect wider trends.

## 5 Conclusions

In conclusion, this study employed multiple analytical models—including mixed-effects and mediation approaches—to examine how the occupational identity of retired Danwei leaders influences post-retirement depression and the mechanisms underlying this relationship. The findings reveal that retired Danwei leaders are significantly more prone to depression than other retirees, with this association mediated by declining physical health and weakened interpersonal relationships. The findings reveal that retired Danwei leaders are significantly more prone to depression than other retirees, with this association mediated by declining physical health and weakened interpersonal relationships.

These results provide empirical support for the theoretical link between role centrality and psychological well-being in later life. By focusing on a distinct occupational subgroup within the Chinese context, this study offers new insights into how former high-status roles may create psychological vulnerabilities after retirement.

In practical terms, the findings underscore the importance of designing targeted interventions that promote physical well-being and sustain social engagement among this group. Moreover, the study highlights the potential mental health benefits of encouraging continued social participation, particularly for retirees from leadership roles. By situating the analysis in the Chinese Danwei context, the study not only reveals the unique challenges of retired leaders but also offers broader implications for societies with hierarchical career systems, thereby contributing to international research and practice in aging and mental health.

## Data Availability

Publicly available datasets were analyzed in this study. This data can be found here: Link to data: http://class.ruc.edu.cn Repository name: China Longitudinal Aging Social Survey (CLASS) Accession number: Not applicable (public dataset, no accession number required).
